# Characteristics of ear fullness and synaptic loss in ear fullness revealed by SV2A positron emission tomographycortical

**DOI:** 10.3389/fnmol.2024.1451226

**Published:** 2024-09-06

**Authors:** En Zhou, Xuping Xiao, Bin Liu, Zhiqiang Tan, JiaYu Zhong

**Affiliations:** ^1^Department of Otolaryngology Head and Neck Surgery, Hunan Provincial People’s Hospital, The First Affiliated Hospital of Hunan Normal University, Changsha, China; ^2^Department of Nuclear Medicine, Xiangya Hospital, Central South University, Changsha, China

**Keywords:** ear fullness, sudden sensorineural hearing loss, synaptic density, cerebral cortex, 18F-SynVesT-1

## Abstract

**Objective:**

Studies on feeling of ear fullness (FEF) related to sudden sensorineural hearing loss(SSNHL) are limited. The mechanisms of FEF are unclear. This study aimed to explore the characteristics and related brain activation of SSNHL with FEF.

**Methods:**

A total of 269 SSNHL patients were prospectively observed and divided into two groups, with FEF and without FEF. Fifteen SSNHL patients with FEF and 20 healthy controls (HCs) were recruited and underwent 18F-SynVesT-1 static PET. Standardized uptake values ratios (SUVr) of 18F-SynVesT-1 were computed between regions of interest.

**Results:**

The occurrence of FEF was not related to the audiogram type or severity of hearing loss. There was a positive correlation between the degree of FEF and the degree of hearing loss. Recovery from FEF was not related to the audiogram shape, the degree of hearing loss or recovery. Fifteen SSNHL patients with FEF had relatively low 18F-SynVesT-1 uptake in the right middle frontal gyrus, right inferior frontal gyrus, right middle temporal gyrus, bilateral parietal lobe sub-gyral and left medial frontal gyrus, as compared with HCs. There was no relatively high 18F-SynVesT-1 uptake in the cerebral cortex.

**Conclusion:**

The occurrence and recovery of FEF in SSNHL patients are not related to the classification, degree and recovery of hearing loss. The 18F-SynVesT-1 uptake in the cerebral cortex of patients experiencing SSNHL and FEF has shown alterations. This indicates that FEF may be related to cortical reorganization after the sudden impairment of unilateral auditory input.

## 1 Introduction

The feeling of ear fullness (FEF) is a common concomitant symptom in patients with sudden sensorineural hearing loss (SSNHL), which is manifested as a sense of blockage, pressure, occlusion etc. It can occur with hearing loss, or it can be the only initial symptom. Sakata and Kato (2006) reported that 83.7% (190/227) of patients with acute sensorineural hearing loss complained of ear fullness. Compared with ear fullness, patients and doctors often pay more attention to more serious symptoms such as hearing loss or tinnitus, vertigo etc. However, even if they have obtained stable hearing, some patients with SSNHL still have a relatively serious sense of ear fullness, which leads to anxiety, boredom and other negative emotions, causing adverse effects on their work, life and sleep (Levo et al., 2014; Zhou et al., 2021). The specific mechanism and characteristics of ear fullness in patients with SSNHL are still unclear, and literature reports are rare (Park et al., 2012; Sakata et al., 2008; Sakata and Kato, 2006; Zhou et al., 2021).

After unilateral auditory deprivation in deafness, the brain undergoes cross-modal compensation, with reorganization of synaptic connections and strength in response to loss of afferent drive (Zeng et al., 2012). Micarelli et al. (2017) showed that idiopathic sudden sensorineural hearing loss (ISSNHL) subjects had hypermetabolism in the right superior and medial frontal gyrus and right anterior cingulate cortex and a relative reduction in fluorodeoxyglucose uptake in the right middle temporal, precentral and postcentral gyrus; left posterior cingulate cortex; left lingual, superior, middle temporal and middle frontal gyrus and left insula. Moreover, glucose consumption in the right anterior cingulate cortex was positively correlated with mean tinnitus handicap inventory (THI). Wang et al. (2021) showed that significantly decreased regional homogeneity (ReHo) in the ipsilateral auditory cortex, as well as increased functional connectivity (FC) between the inferior parietal gyrus and the auditory cortex were found in the idiopathic sudden sensorineural hearing loss (ISSNHL) with vertigo groups. To our knowledge, there is currently no study on cortical synaptic plasticity in SSNHL with FEF.

Synaptic vesicular glycoprotein 2A (SV2A) is an integral glycoprotein in the membrane of synaptic vesicles located in the presynaptic end, which is involved in vesicle trafficking and exocytosis and widely distributed throughout the brain (Mikkelsen et al., 2023). The novel SV2A positron emission tomography (PET) radiotracer 18F SynVesT-1 can measure synaptic density and has been widely used in the assessment of synaptic loss and lesion localization in various neuropsychiatric diseases (Carson et al., 2022). Unlike 18F-FDG and fMRI, 18F-SynVesT-1 directly targets synapses, quantifies synaptic density and is particularly suitable for detecting changes in cortical synapses in the early stages of disease.

We hypothesized that specific synaptic density signatures are present in SSNHL with FEF. In this study, we observed the characteristics and prognosis of ear fullness in SSNHL. Additionally, based on 18F-SynVesT-1-PET synaptic density imaging, we will investigate the relationship between ear fullness and synaptic reorganization after auditory deprivation in patients with SSNHL.

## 2 Materials and methods

### 2.1 Inclusion and exclusion criteria

This study used a prospective study protocol. Patients with unilateral SSNHL were included. Regarding complaint of ear fullness, they were divided into two groups: with and without ear fullness. According to the frequency of hearing loss, they were divided into a low-frequency decline type (hearing loss at frequencies below 1,000 Hz (inclusive) and hearing loss at least at 250 and 500 Hz ≥ 20 dBHL), a high-frequency decline type (hearing loss at frequencies above 2,000 Hz (inclusive), hearing loss at least at 4,000 and 8,000 Hz ≥ 20 dBHL) and an all-frequency decline type (hearing loss at all frequencies ≥20 dBHL).

The inclusion criteria were as follows: 1. Fulfilment of the diagnostic criteria of SSNHL in the 2015 Chinese Guidelines for the diagnosis and treatment of SSNHL (2015), including sudden sensorineural hearing loss of unknown cause within 72 h, hearing loss ≥20 dBHL at a minimum of two adjacent frequencies. 2. Presence of a single ear disease. The time from the onset to the hospital visit was ≤14 days. No treatment was given during the period from the onset to the hospital visit. 3. Presence of ear fullness accompanied by the appearance of SSNHL, or the initial symptom of the disease. 4. Age between 18 and 65 years and the ability to cooperate in completing audiological and imaging examinations and the subjective severity assessment scale of ear fullness.

The exclusion criteria were the following: 1. Presence of external and middle ear diseases, superior semi-circular canal fissure syndrome, endolymphatic sac tumour, Meniere’s disease, acoustic nerve space occupying, nasal sinus related diseases, parapharyngeal space diseases, temporomandibular joint disorders, rheumatic immune-related diseases, cardiovascular and cerebrovascular diseases, psychosis, diabetes and other space-occupying diseases of the whole body. 2. History of SSNHL, Meniere’s disease, ear surgery or migraine. 3. Inability to cooperate with follow-up.

### 2.2 Follow-up

Two hundred ninety-eight patients with unilateral SSNHL hospitalised in the Department of Otolaryngology Head and Neck Surgery of Hunan Provincial People’s Hospital from June 2015 to September 2022 were included in the study. According to the above inclusion and exclusion criteria, 29 patients who did not meet the conditions were excluded, including four patients with bilateral diseases successively, one patient with a space-occupying lesion of the auditory nerve found on MRI of the internal auditory canal, four patients with chronic sinusitis, two patients with a previous history of SSNHL, four patients with cardiovascular and cerebral vascular diseases and 14 patients unable to cooperate in completing the examination or follow-up. Finally, 269 patients were followed up.

All patients in this study were followed up by way of outpatient return visit and WeChat communication for 1 month. Students specially designated by the department contacted any patients who failed to attend timely outpatient follow up. The results of pure tone hearing threshold and VAS score of ear fullness after 1 month of treatment are the final recovery results.

### 2.3 Examination and treatment

All patients were examined and treated according to the recommendations of China’s 2015 Guidelines for the Diagnosis and Treatment of SSNHL (Editorial Board of Chinese Journal of Otorhinolaryngology Head and Neck Surgery et al., 2015). All patients completed the subjective severity assessment scale of ear fullness. The following examinations were completed: otoendoscope, pure tone hearing threshold, acoustic conduction anti-eustachian tube function examination, otoacoustic emission (TEOAE, DPOAE), auditory evoked potential (ABR), Audio Steady-State Response (ASSR), plain and enhanced MRI of the internal auditory canal and temporal bone CT.

All patients were treated immediately after hospitalisation, mainly including an intravenous drip of dexamethasone sodium phosphate injection at 10 mg/day; the dosage was reduced to 5 mg/day after 7 days, and the drug was stopped after 3 days of continuous use. Ginkgo damole injection (*Ginkgo biloba* leaf extract) was administered at 20 mL/day and discontinued after 10 days of continuous use. Oral mecobalamin (nutritional nerve) was administered at 0.5 mg/dose, 3 times/day, and discontinued after 10 days of continuous use. Patients with severe tinnitus were treated with an intravenous drip of 2% lidocaine injection at 20 ml/day for 7 days. The intake of normal saline was limited in patients with SSNHL of the low-frequency descent type. Patients with poor efficacy were treated with a hyperbaric oxygen supplement and tympanic hormone injection. During the treatment, the hearing was rechecked twice a week. If the patient recovered, the treatment was terminated early.

### 2.4 PET imaging and data analysis

Fifteen SSNHL patients with ear fullness and 20 healthy controls (HCs) were recruited. All participants underwent 18F-SynVesT-1 static PET imaging and magnetic resonance imaging (MRI). The compound 18F-SynVesT-1 was synthesized using previously described methods (Li et al., 2019; Naganawa et al., 2021). All participants did not take drugs targeted to SV2A for at least 24 h before their scans. Static PET images were acquired in three dimensions for 30 min, starting at ~60 min after intravenous injection of 18F-SynVesT-1 (3.7 MBq/kg for 1 min). PET/computed tomography (CT) images were acquired by a Discovery Elite PET/CT scanner (GE Healthcare). Participants were placed in the PET scanner so that slices were parallel to the canthomeatal line. Data were reconstructed with a 3D VUE Point (GE Healthcare) ordered-subset expectation maximization algorithm with two iterations and 23 subsets as described previously (Tang et al., 2022). To assist co-registration with PET images, T1-weighted structural MRI was conducted for all patients. PET images were coregistered to the participant’s T1-weighted MR image using statistical parametric mapping software (SPM12, University College London) and Computational Anatomy Tool-box (CAT12; Tzourio-Mazoyer et al., 2002). The transformation parameters determined by MRI spatial normalization were then applied to the co-registered 18F-SynVesT-1 PET images for PET spatial normalization. Semiquantitative analysis was performed for all PET data. Visual assessment of PET images was undertaken by a single operator. Standardized uptake values (SUVs) were calculated for all regions of interest (ROIs), and standardized uptake values ratio (SUVr) with the centrum semiovale (CS) as a reference region was calculated for interpatient comparisons (Finnema et al., 2020). Comparisons between groups were performed using the Mann–Whitney U test of SPM. Sex and age at PET with tinnitus and time of education were used as covariates in comparisons between SSNHL patients and HCs. The height threshold of synaptic density changes was set at *p* < 0.001 [*p* < 0.05 familywise error (FWE) corrected at cluster]. After data were preprocessed using SPM, significant clusters were visualized, reported and anatomically labelled using the xjView MATLAB toolboxes.

### 2.5 Grades of hearing loss and efficacy evaluation

Hearing loss was graded according to WHO-1997 (Smith, 1997) as (1) extremely severe hearing loss: PTA ≥81 dBHL; (2) severe hearing loss: 61–80 dBHL; (3) moderate hearing loss: 41–60 dBHL; (4) slight hearing loss: 26–40 dBHL; (5) normal hearing: PTA ≤25 dBHL. In the scaling-out cases, calculations were performed by adding 5 dB to the maximum level of sound generated by the audiometer. All frequency hearing thresholds were calculated for the all-frequency hearing loss type, and the damaged frequency was calculated for the high- and low-frequency hearing loss types.

Efficacy evaluation (2015): 1. Cured: hearing is completely restored to the level before the current illness or to the normal ear. 2. Remarkable effect: the average hearing threshold of the damaged frequency is improved by ≥30 dB. 3. Effective: The average hearing threshold of the damaged frequency is improved by 15–30 dB. 4. Ineffective: the improvement is less than 15 dB.

### 2.6 Grades of ear fullness and efficacy evaluation

Grades of ear fullness: According to a previous experience (Zhou et al., 2021), the severity of ear fullness was graded by visual analogue scale (VAS) score: “Slight” (1–2 points); Slight ear fullness, hazy feeling, not affecting mood, work and social life, and a weak desire for treatment. “Moderate” (3–4 points); There is obvious sense of ear fullness and occlusion, which can cause boredom, without affecting work and social life, and has a desire for treatment. “Pretty” (5–6 points): There is an obvious sense of ear fullness, blockage, and pressure, which can cause depression and anxiety, affect work and social life, not affect sleep, and has a strong desire for treatment. “Serious” (7–8 points): There is a very obvious sense of ear fullness and distension, which causes boredom, irritability, and anxiety, has a significant impact on work and social life, affects sleep, and has a strong desire for treatment. “Extremely Serious” (9–10 points): Ear fullness causes severe discomfort, irritability, anxiety, and serious impact on work, social life, and sleep. The desire for treatment is higher than the hearing loss, which is very strong.

Efficacy evaluation: Cured: VAS score was 0; Remarkable effect: VAS score improved by more than 4 points or less than 1/2 of the initial score; Effective: VAS score improved by more than 2 points or more or equal to 1/2–2/3 of the initial score; Ineffective: VAS score improved by less than 2 points or greater than 2/3 of the initial score or recurrence within 1 week.

### 2.7 Clinical data analysis

SPSS v 23.0 software was used to analyse the clinical data. The rank sum test was used for counting data of ordered classification. Chi-square test for fourfold table data, and if the conditions for the chi-square test are not met, Fisher’s exact probability method can be used. For count data that meets the assumptions of normal distribution and homogeneity of variance, a t-test is used. Spearman rank correlation analysis was used for two-way ordered rank data. Results were considered statistically significant at *p* < 0.05.

## 3 Results

### 3.1 Patient information

Two hundred sixty-nine patients with unilateral SSNHL were included, 128 males (47.6%) and 141 females (52.4%). One hundred forty-six cases (54.3%) in the group had ear fullness, and 123 cases (45.7%) in the group did not have ear fullness. There was no difference between the two groups in age, gender, deafness side, course of disease, type of hearing loss, degree of hearing loss and ABR elicitation (*p* > 0.05; Table 1 and Supplementary Tables 1–2).

**Table 1:** Comparison of the general characteristics of the two groups of patients.

	With ear fullness	Without ear fullness	Statistical test value	*p*-value
Total number(*n*)	146	123		
Gender(Male/Female; *n*)	66/80	62/61	*χ*^2^ = 0.724	*p* = 0.395
Side (left/right; *n*)	74/72	66/57	*χ*^2^ = 0.237	*p* = 0.627
Age(y)	41.92 ± 14.68	42.27 ± 14.63	*t* = 0.195	*p* = 0.846
Course of disease(d)	4.05 ± 1.97	4.11 ± 2.12	*t* = 0.240	*p* = 0.810
Type of hearing loss			*Z* = 1.434	*p* = 0.151
Low-frequency descent type	39	30		
High-frequency descent type	29	26		
All-frequency descent type	78	67		
Degree of hearing loss(*n*)			*Z* = 0.405	*p* = 0.686
Slight	13	10		
Moderate	45	36		
Severe	51	44		
Extremely severe	37	33		
ABR(*n*)			*χ*^2^ = 0.064	*p* = 0.801
Normal	94	81		
Abnormal	52	42		

### 3.2 Relationship between the grade of ear fullness and degree of hearing loss

There was a positive correlation between the grade of ear fullness and the degree of hearing loss in patients with SSNHL (r_s_ = 0.442, *p* = 0.000; Table 2).

**Table 2:** Relationship between the grade of ear fullness and the degree of hearing loss.

Grade of ear fullness	Degree of hearing loss [*n* (%)]				Total
	Slight	Moderate	Severe	Extremely severe	
Slight (0–2)	7 (4.8)	12 (8.2)	4 (2.7)	2 (1.4)	25 (17.1)
Moderate (3–4)	6 (4.1)	23 (15.8)	19 (13.0)	15 (10.3)	63 (43.2)
Pretty (5–6)	0 (0.0)	8 (5.5)	19 (13.0)	7 (4.8)	34 (23.3)
Serious (7–8)	0 (0.0)	2 (1.4)	9 (6.2)	13 (8.9)	24 (16.4)
Total	13 (8.9)	45 (30.8)	51 (34.9)	37 (25.3)	146 (100.0)

### 3.3 Relationship between hearing recovery and ear fullness

Hearing recovery: After 1 month of treatment, the total efficacy rate of hearing recovery was 74.7% (201/269). There was no statistical difference in hearing recovery between the patients with and without ear fullness (*Z* = 0.296, *p* = 0.767; Table 3).

**Table 3:** Relationship between hearing recovery and ear fullness.

Group	Total (*n*)	Hearing recovery [*n* (%)]				
		Cured	Remarkable effect	Effective	Ineffective	Total effective rate
With ear fullness	146	51 (34.9)	31 (21.2)	27 (18.5)	37 (25.3)	109 (74.7)
Without ear fullness	123	48 (39.0)	20 (16.3)	24 (19.5)	31 (25.2)	92 (74.8)

Hearing recovery in patients with different subtypes of SSNHL: The total efficacy rate of hearing recovery in patients with different subtypes of SSNHL was 92.8% (64/69) in low-frequency descent type, 67.3% (37/55) in high-frequency descent type, and 69.0% (100/145) in all-frequency descent type. The difference of hearing recovery among different subtypes was statistically significant (H = 48.75, *p* = 0.000). There was no statistical difference in the hearing recovery of patients with low-frequency descent, high-frequency descent, and all-frequency descent SSNHL in the groups with and without ear fullness (*Z* = −0.737, −0.368, −0.029 and *p* = 0.461, 0.713, 0.977, respectively; Table 4).

**Table 4:** Relationship between hearing recovery and ear fullness in patients with SSNHL of various subtypes.

Group		Total (*n*)	Hearing recovery [*n* (%)]				
			Cured	Remarkable effect	Effective	Ineffective	Total effective rate
Low-frequency descent type	With ear fullness	39	28 (71.8)	4 (10.3)	4 (10.3)	3 (7.7)	36 (92.3)
	Without ear fullness	30	24 (80.0)	2 (6.7)	2 (6.7)	2 (6.7)	28 (93.3)
High-frequency descent type	With ear fullness	29	6 (20.7)	5 (17.2)	9 (31.0)	9 (31.0)	20 (69.0)
	Without ear fullness	26	8 (30.8)	4 (15.4)	5 (19.2)	9 (34.6)	17 (65.4)
All-frequency descent type	With ear fullness	78	17 (21.8)	22 (28.2)	14 (17.9)	25 (32.1)	53 (67.9)
	Without ear fullness	67	16 (23.9)	14 (20.9)	17 (25.4)	20 (29.9)	47 (70.1)

### 3.4 Relationship between recovery of ear fullness and hearing loss

Recovery of ear fullness: 43.2% (63/146) of patients with ear fullness disappeared after 1 week of treatment, and the total efficacy rate was 88.4% (129/146). After 1 month of treatment, 88.4% (129/146) of patients were cured of ear fullness, and the total efficacy rate was 95.2% (139/146). The effect after 1 month of treatment was better than that after 1 week of treatment (*Z* = 7.778, *p* = 0.000; Table 5).

**Table 5:** Recovery of ear fullness.

Group	Total (*n*)	Recovery of ear fullness [*n* (%)]				Total effective rate
		Ineffective	Effective	Remarkable effect	Cured	
After 1 week of treatment	146	17 (11.6)	24 (16.4)	42 (28.8)	63 (43.2)	129 (88.4)
After 1 month of treatment	146	7 (4.8)	2 (1.4)	8 (5.5)	129 (88.4)	139 (95.2)

Recovery of ear fullness in patients with different subtypes of SSNHL: After 1 month of treatment, the total efficacy rate of recovery of ear fullness was 97.4% (38/39) for low-frequency descent type, 93.1% (27/29) for high-frequency descent type, and 94.9% (74/78) for full frequency descent type. There was no statistical difference among the three groups (H = 0.890, *p* = 0.641; Table 6).

**Table 6:** Recovery of ear fullness in SSNHL patients with different subtypes.

Group	Total (*n*)	Recovery of ear fullness [*n* (%)]				Total effective rate
		Ineffective	Effective	Remarkable effect	Cured	
Low-frequency descent type	39	1 (2.6)	0 (0.0)	2 (5.1)	36 (92.3)	38 (97.4)
High-frequency descent type	29	2 (6.9)	1 (3.4)	1 (3.4)	25 (86.2)	27 (93.1)
All-frequency descent type	78	4 (5.1)	1 (1.3)	5 (6.4)	68 (87.2)	74 (94.9)

Recovery of ear fullness in SSNHL patients with different degree of hearing loss: There was no statistical difference in the recovery of ear fullness in SSNHL patients with different hearing thresholds (H = 2.065, *p* = 0.559; Table 7).

**Table 7:** Recovery of ear fullness in SSNHL patients with different degree of hearing loss.

Degree of hearing loss	Total (*n*)	Recovery of ear fullness [*n* (%)]				Total effective rate
		Ineffective	Effective	Remarkable effect	Cured	
Slight	13	1 (7.7)	0 (0.0)	0 (0.0)	12 (92.3)	12 (92.3)
Moderate	45	0 (0.0)	1 (2.2)	2 (4.4)	42 (93.3)	45 (100)
Severe	51	4 (7.8)	1 (2.0)	2 (3.9)	44 (86.3)	47 (92.2)
Extremely severe	37	2 (5.4)	1 (2.7)	3 (8.1)	31 (83.8)	35 (94.6)

Recovery of ear fullness after 1 month of treatment is not related to the improvement of hearing (rs = 0.146, *p* = 0.079; Table 8).

**Table 8:** Relationship between recovery of ear fullness and hearing recovery.

Recovery of ear fullness	Recovery of hearing loss [*n* (%)]				Total
	Ineffective	Effective	Remarkable effect	Cured	
Ineffective	3 (2.1)	2 (1.4)	2 (1.4)	0 (0.0)	7 (4.8)
Effective	1 (0.7)	0 (0.0)	0 (0.0)	1 (0.7)	2 (1.4)
Remarkable effect	3 (2.1)	2 (1.4)	0 (0.0)	3 (2.1)	8 (5.5)
Cured	30 (20.5)	23 (15.8)	29 (19.9)	47 (32.2)	129 (88.4)
Total	37 (25.3)	27 (18.5)	31 (21.2)	51 (34.9)	146 (100%)

### 3.5 Synaptic density changes in SSNHL patients with ear fullness

#### 3.5.1 Demographic and clinical features of subjects

As shown in Table 9, SSNHL patients with ear fullness and HCs were well matched for age, sex and education. There was no significant difference among groups in sex distribution, age and various clinical indicators (*p* > 0.05). The 18F-SynVesT-1 injection was well tolerated, and no subjective or objective adverse effects were detected.

**Table 9:** Demographic and clinical features of subjects.

Group	Total	With ear fullness	Health controls	Statistical test value	*p*
*N*	35	15	20	-	-
Sex, male/female	19/16	8/7	11/9	-	*p* = 1.000
Age, years ± SD	41.29 ± 13.33	41.87 ± 13.87	40.85 ± 12.26	*t* = 0.230	*p* = 0.450
Education, years ± SD	11.00 ± 1.85	10.93 ± 2.01	11.05 ± 1.76	*t* = 0.188	*p* = 0.426
Side (left/right; *n*)	7/8	7/8	-	-	-
Time from onset to PET, days ± SD	3.93 ± 1.83	3.93 ± 1.83	-	-	-
Degree of hearing loss (Severe/Extremely severe)	5/10	5/10	-	-	-

#### 3.5.2 Synaptic density changes

**Figure 1:**
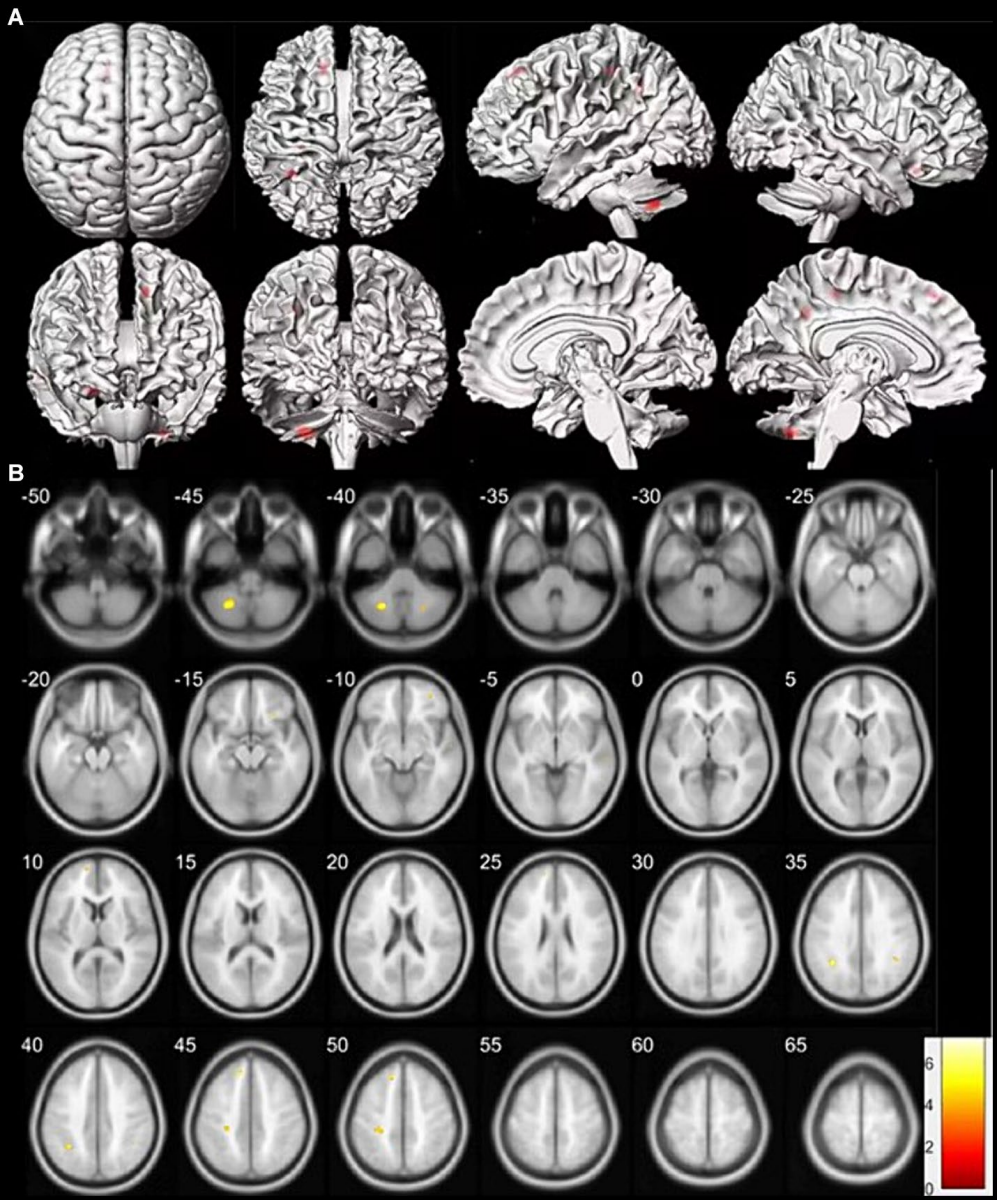
Clusters of synaptic change in SSNHL patients with ear fullness versus healthy controls (HCs). **(A)** The clusters of synaptic changes are shown on a three-dimensional brain template. The region of synaptic change displayed in red. **(B)** The clusters of synaptic changes are shown on a magnetic resonance imaging template. Low intake displayed in cool colors. Data were analysed at a height threshold of *p* < 0.001 and were cluster-level corrected for familywise error at *p* < 0.05.

On an individual visual level, as diagnosed in routine clinical practice using 18F-SynVesT-1-PET imaging, all patients had a visually normal 18F-SynVesT-1-PET. Voxel-based group analysis showed that SSNHL patients with ear fullness had relatively low 18F-SynVesT-1 uptake in the right middle frontal gyrus, right inferior frontal gyrus, right middle temporal gyrus, bilateral parietal lobe sub-gyral and left medial frontal gyrus, as compared to that of HCs (the height threshold was set at *p* < 0.001, *p* < 0.05 FWE corrected at cluster). There was no relatively high 18F-SynVesT-1 uptake in the cerebral cortex. Coordinate and regional details are presented in Figure 1 and Table 10.

**Table 10:** Location and peaks of significant synaptic density reduction in SSNHL patients with ear fullness compared with that of HCs.

*p* (FWE corrected)	Cluster extent	*Z*-score	Peak coordinates (*x*, *y*, *z*), mm			Lobe	Cortical region	BA
0.000	13	4.46	34	50	−8	Right Frontal Lobe	Middle Frontal Gyrus	
	22	4.30	24	24	−16	Right Frontal Lobe	Inferior Frontal Gyrus	47
	28	4.23	58	−24	−6	Right Temporal Lobe	Middle Temporal Gyrus	21
	57	4.03	−32	−50	36	Left Parietal Lobe	Sub-Gyral	
	29	3.60	−14	34	48	Left Frontal Lobe	Medial Frontal Gyrus	
	42	3.44	−30	−30	48	Right Parietal Lobe	Sub-Gyral	

BA, Brodmann area; FWE, familywise error; HC, healthy control.

## 4 Discussion

Of SSNHL patients, 40.2–83.7% were afflicted by ear fullness (Sakata and Kato, 2006; Wang et al., 2015; Zhou et al., 2021). FEF often occurs in the ear with hearing loss, and a few patients can experience mild ear fullness in the healthy ear. In this study, the incidence of ear fullness was 54.3% (146/269), and the male-to-female ratio was 0.825:1 (66/80). Seventy-four cases had left ear fullness, and 72 cases had right ear fullness. The healthy ears of four patients had mild to moderate ear fullness, and there was no statistical difference between males and females. These results are consistent with literature reports (Wang et al., 2015). There was no statistical difference in gender, age, auditory evoked potential results, audiogram type and degree of hearing loss between the patients with and without ear fullness, suggesting that these factors had nothing to do with the presence of ear fullness and were not predictive factors of ear fullness. Ear fullness is a subjective sensation, although common in Eustachian tube dysfunction, Meniere’s disease, and other conditions, there is a lack of methods for assessing the severity of ear fullness in clinical practice. Zhai et al. (2013) used a Visual Analog Scale (VAS) rating scale (1–10 points) to assess the severity of ear fullness in patients with Meniere’s disease, but no detailed description of the assessment was provided. In our preliminary research, (Zhou et al., 2021) we used VAS scoring to classify ear fullness into slight, moderate, pretty, serious, extremely serious based on the patient’s description of ear fullness, its impact on emotions, work and social interactions, its impact on sleep, and the patient’s desire for treatment. The severity of ear fullness of the subjects in this study was predominantly moderate (43.2%), followed by moderate to pretty severe (23.3%), indicating that the mood, work and social activities of the patients were affected. No patients had extremely serious ear fullness, and only 16.4% (24/146) of patients had serious ear fullness, indicating that ear fullness rarely affects patients’ sleep, and few patients have a strong desire for treatment. Spearman correlation analysis showed that there was a positive correlation between the grade of ear fullness and the degree of hearing loss (rs = 0.442). Sakata and Kato (2006) showed that the occurrence of ear fullness in patients with acute sensorineural hearing loss was not related to the type of hearing loss, but evaluation of the degree of ear fullness was not mentioned in this study. The higher the hearing threshold, the greater the impact on the patient’s mood and life. Hearing impairment and the emotional changes caused by it may also aggravate the feeling of ear fullness, so the relationship between the hearing threshold and ear fullness needs further study. There was no statistical difference between the total efficacy rate of hearing recovery in the groups with ear fullness (74.7%) and without ear fullness (74.8%), indicating that ear fullness was not a relevant prognostic marker in patients with SSNHL. After 1 month of treatment, the rate of recovery for ear fullness was 88.4%, and the overall efficacy rate was 95.1%. With the recovery of hearing, most patients experienced a disappearance of ear fullness. However, many patients who did not fully regain their hearing also experienced a disappearance of ear fullness. This suggests that the symptoms of ear fullness have a good self-healing nature, and there is no correlation between the improvement of ear fullness and the recovery of hearing.

The mechanism causing ear fullness in SSNHL is still unclear. Zheng et al. (2019) found that the incidence of endolymphatic hydrops was 68.0%, higher than the 34.8% found in the healthy ear in patients with SSNHL. Okazaki et al. (2017) showed that there was no statistical difference in the incidence of endolymphatic hydrops in the affected ear (66%) compared with that in the healthy ear (52%) in patients with SSNHL. Whether patients with SSNHL have endolymphatic hydrops is still controversial. Ear fullness is also not a characteristic symptom of endolymphatic hydrops (Levo et al., 2014; Zhai et al., 2013). Sakata et al. (2012) found that there was a statistical difference between the tympanic membrane minimum sensory threshold for air pressure loading (MSTAP and daPa) of the affected ear and the normal ear at the first medical examination. The MSTAP measured at the first medical examination also differed from that measured at the time a steady audiogram was obtained. That is to say, the somatosensory regulation ability of the tympanic membrane in patients with SSNHL has changed, which may be related to the generation of ear fullness, but the specific mechanism is unclear. Our research found that the occurrence of ear fullness in SSNHL is not related to the frequency or severity of hearing loss. Ear fullness has a high rate of self-healing and is not related to the recovery of hearing. This suggests that ear fullness may not be caused by organic lesions in the inner ear but may be due to some functional factor, similar to tinnitus.

Few PET studies have focused on cortical reorganization after SSNHL. Micarelli et al. (2017) showed that ISSNHL subjects had hypermetabolism in the right superior and medial frontal gyrus as well as in the right anterior cingulate cortex and a relative reduction in fluorodeoxyglucose uptake in the right middle temporal, precentral and postcentral gyrus; left posterior cingulate cortex; left lingual, superior, middle temporal and middle frontal gyrus and left insula. This suggests that synaptic plasticity of the auditory cortex and surrounding related areas begins in the early stage of ISSNHL. Verger et al. (2017) showed that late-onset deafness patients were found to have decreased metabolism in pre- and post-central areas, the cingulum, the right inferior parietal gyrus and the striatum on both sides, while increased metabolism was found in the prefrontal areas, the pre- and post-central areas, the cingulum and the left inferior parietal gyrus. Lee et al. (2003) showed that postlingually deaf patients had a relative reduction in fluorodeoxyglucose uptake in both anterior cingulate gyri [Brodmann area 24 (BA24)] and superior temporal cortices (BA41, BA42) and in the right parahippocampal gyrus. No area showed a significant increase in metabolism in deaf patients with the same threshold. Okuda et al. (2013) showed that glucose metabolism in postlingual deaf patients was lower in the right superior temporal gyrus, both middle temporal gyri, left inferior temporal gyrus, right inferior lobulus parietalis, right posterior cingulate gyrus, and left insular cortex than that of the control subjects. This suggests that in the mature brain, auditory deprivation decreased neuronal activity transiently in primary auditory and auditory-related cortices, and, over time, functional reorganization likely takes place in the auditory cortex. This study showed that SSNHL patients with ear fullness had relatively low 18F-SynVesT-1 uptake in the right middle frontal gyrus, right inferior frontal gyrus, right middle temporal gyrus, bilateral parietal lobe sub-gyral and left medial frontal gyrus, as compared to that of HCs. There was no relatively high 18F-SynVesT-1 uptake in the brain lesions of SSNHL patients with ear fullness. It was suggested that remodelling in synaptic density and intensity in the auditory cortex and surrounding related areas occur in the early stages of SSNHL, consistent with previous research. Cortical reorganization in patients with ear fullness is mainly manifested in the frontal lobes parietal lobes, and right middle temporal gyrus, in which the frontal lobe has extensive communication fibres related to memory, judgment, abstract thinking, speech expression, emotions and impulsive behaviour (Chayer and Freedman, 2001). The right middle temporal gyrus is an important audiovisual integration area, and it also plays a key role in language processing, cognition, emotion, and memory (Scheliga et al., 2023). The parietal lobe sub-gyral is an important multisensory integration area responsible for integrating information from different sensory systems (such as visual, auditory, and tactile; Kassuba et al., 2020). The parietal lobe sub-gyral contains the primary cortical somatosensory area, which helps to explain and perceive tactile input, pressure and perception of the weight, mass and composition of objects (Pellicer-Morata et al., 2023). Therefore, following unilateral auditory hearing loss, there is a downregulation and reorganization of cortical neural inputs involved in auditory information integration and processing. The early reorganization of cortical function following unilateral hearing loss may have a broad impact on patients’ perception, cognition, and emotions. This may partially explain the appearance of ear fullness during the sudden impairment of unilateral auditory input. The reorganization of the cortical somatosensory area in the parietal lobe sub-gyral may affect the patient’s perception and understanding of tactile and pressure sensation information, “mistakenly” attributing auditory decline to ear fullness. Our research has found that ear fullness has a high rate of self-healing. In addition, the recovery rate of ear fullness after 1 month of treatment is significantly higher than that after 1 week of treatment. This suggests that the brain may have corrected this “erroneous cognition” in the later stage of cortical reorganization. This also tells us that for patients experiencing ear fullness after SSNHL, it is beneficial to allow a certain amount of time for self-recovery of the ear fullness. At the same time, informing patients of the clinical characteristics of ear fullness and its favorable prognosis can help alleviate their anxiety and other negative emotions.

Of course, our research also has certain limitations. Although the reorganization of cortical somatosensory regions in the early stage in patients with SSNHL and ear fullness may to some extent suggest potential changes in tactile and pressure perception. However, due to the limited sample size included, we were unable to compare patients with SSNHL without ear fullness separately from those with SSNHL and ear fullness to a healthy control group. We are also unable to conduct comparative analysis of 18F-SynVesT-1 uptake in these areas among different subtypes of sudden deafness patients. In addition, PET imaging can only provide static information and cannot observe dynamic changes in brain activity, limiting a deeper understanding of the relationship between ear fullness and brain remodeling. In the future, we could further expand the sample size and combine techniques such as fMRI, high-density EEG, to explore cortical reorganization patterns in different subtypes of sudden deafness patients, as well as the dynamic correlation between ear fullness and cortical reorganization.

## 5 Conclusion

Our study shows three characteristics of ear fullness in patients with SSNHL. First, the occurrence of ear fullness is not related to the classification and grading of hearing loss, and the severity of ear fullness is related to the degree of hearing loss. Second, the recovery of ear fullness was better, and there was no correlation with the classification, severity and recovery of hearing loss. Third, the relative changes in 18F-SynVesT-1 uptake found in these brain regions in SSNHL highlight new aspects of cerebral rearrangement, which may explaining the appearance of ear fullness during the sudden impairment of unilateral auditory input.

## Data availability statement

The original contributions presented in the study are included in the article/Supplementary material, further inquiries can be directed to the corresponding author.

## Ethics statement

The studies involving humans were approved by Institutional Review Board of Hunan Provincial People’s Hospital, China (201577, May 25, 2015). The studies were conducted in accordance with the local legislation and institutional requirements. The participants provided their written informed consent to participate in this study. Written informed consent was obtained from the individual(s) for the publication of any potentially identifiable images or data included in this article.

## Author contributions

EZ: Conceptualization, Data curation, Funding acquisition, Writing – original draft, Writing – review & editing. XX: Visualization, Writing – review & editing, Investigation, Resources, Conceptualization, Project administration. BL: Investigation, Methodology, Resources, Writing – review & editing. ZT: Conceptualization, Project administration, Supervision, Visualization, Writing – review & editing. JZ: Investigation, Visualization, Writing – review & editing, Data curation, Software, Conceptualization.
